# Prognostic value of differentiation status in gastric cancer

**DOI:** 10.1186/s12885-018-4780-0

**Published:** 2018-09-03

**Authors:** Fan Feng, Jinqiang Liu, Fei Wang, Gaozan Zheng, Qiao Wang, Shushang Liu, Guanghui Xu, Man Guo, Xiao Lian, Hongwei Zhang

**Affiliations:** 1 Division of Digestive Surgery, Xijing Hospital of Digestive Diseases, Fourth Military Medical University, 127 West Changle Road, Xi’an, 710032 Shaanxi China; 2 Cadre’ s sanitarium, 62101 Army of PLA, 67 Nahu Road, Xinyang, 464000 Henan China; 3 Department of General Surgery, No. 534 Hospital of PLA, Yingzhou Road, Luoyang, 471000 Henan China; 4 Department of General Surgery, No. 91 Hospital of PLA, 239Gongye Road, Jiaozuo, 454000 Henan China

**Keywords:** Gastric cancer, Differentiation status, Clinicopathological characteristics, Prognosis

## Abstract

**Background:**

Up to date, investigation of the prognostic value of differentiation status mainly focused on signet ring cell and mucinous gastric cancer. Thus, the present study aims to investigate the clinicopathological features and prognosis of gastric cancer patients with well, moderately and poorly differentiation status.

**Methods:**

From September 2008 to March 2015, a total of 3090 gastric cancer patients treated with radical D2 gastrectomy were enrolled in the present study. Clinicopathological characteristics and prognosis of gastric cancer patients with well, moderately and poorly differentiation status were analyzed.

**Results:**

There were 2422 male (78.4%) and 668 female (21.6%). The median age was 58 (20–90) years. There were 370 (12.0%) well differentiated tumors, 836 (27.0%) moderately differentiated tumors and 1884 (61.0%) poorly differentiated tumors. Well and moderately differentiation status were associated with older age, male gender, smaller tumor, shallower invasion, less lymph node involvement and earlier tumor stage (all *p* < 0.001). Inversely, poorly differentiation status was associated with younger age, female gender, larger tumor, deeper invasion, more lymph node involvement and later tumor stage (all *p* < 0.001). With respect to prognosis, well differentiation status was associated with favorable overall survival and poorly differentiation status was associated with unfavorable overall survival (*p* < 0.001). However, after matching with age, tumor size, T and N stage, there was no significant difference among the overall survival of the three groups (*p* = 0.415).

**Conclusions:**

Well, moderately and poorly differentiation status was significantly associated with clinicopathological features of gastric cancer patients. However, it was not associated with the prognosis of gastric cancer patients.

## Background

Despite the decreasing overall worldwide incidence of gastric cancer in the recent decades, it is still the fifth most common malignant tumors and the third leading cause of cancer-associated death in the world [1]. Furthermore, it is the second commonest cancer in China [2]. Surgical resection with extended lymph node clearance remains the only curative option for non-metastatic gastric cancer [3–5]. Although with the significant improvement in surgical techniques and adjuvant therapy, the prognosis of gastric cancer is still not promising [6]. Thus, the prognostic risk factors for gastric cancer have been widely investigated, and age, tumor location, tumor size, tumor depth and lymph node metastasis, etc. [4, 7, 8] have already been demonstrated to be associated with the prognosis of gastric cancer patients previously.

Up to date, the investigation of the prognostic value of differentiation status mainly focused on signet ring cell (SRC) and mucinous carcinoma, which were classified as undifferentiated type according to the Japanese classification of gastric carcinoma [9]. However, the prognostic significance of SRC and mucinous gastric carcinoma are still controversial [10–14]. Previously, we also investigated the clinicopathological features and prognostic value of mucinous gastric cancer, and found that the prognosis of mucinous tumor was comparable with non-mucinous tumor [15]. However, the clinicopathological characteristics and prognosis of patients with well, moderately and poorly differentiated gastric cancer have not been reported yet. Thus, the present study aims to investigate the clinicopathological features and prognosis of gastric cancer patients with well, moderately and poorly differentiation status.

## Methods

This study was performed in the Division of Digestive Surgery, Xijing Hospital of Digestive Diseases. From September 2008 to March 2015, a total of 3090 consecutive gastric cancer patients were enrolled in the present study. All patients were treated with radical D2 gastrectomy [16] and regular follow up. This study was approved by the Ethics Committee of Xijing Hospital, and written informed consent was obtained from all patients before surgery.

Clinicopathological data including age, gender, tumor location, tumor size, differentiation status, tumor depth, lymph node metastasis and tumor stage were collected. Patients were divided into three groups according to differentiation status: well, moderately and poorly differentiation. The patients were followed up till November 2016.

Data were processed using SPSS 22.0 for Windows (SPSS Inc., Chicago, IL, USA). Discrete variables were analyzed using Chi-square test or Fisher’s exact test. We respectively matched the 1884 patients in poorly differentiation group and 836 patients in moderately differentiation group to the 370 patients who in well differentiation group with a 1:1 matched ratio. The gmatch macro of SAS 9.2 (SAS institute, Cary, NC, USA) written by Erik Bergstralh & Jon Kosanke (http://bioinformaticstools.mayo.edu/research/gmatch/) was used to execute this pair matching process. The principle of this macro is computerized matching of cases to controls using the greedy matching algorithm with a fixed number of controls per case. Controls may be matched to cases using one or more factors (X’s). In this study, we set patients in well differentiation group as cases, and patients in moderately or poorly group as controls. The matching condition/factors we set among the four groups based on the following three parameters: age (±10 years), tumor size (±1 cm), T stage and N stage. The study endpoint was OS (Overall survival), the time to event was computed from the date of surgery to the date when the death (any cause) was recorded. Overall survival curves were estimated by the Kaplan-Meier method and comparison between survival curves was performed using the log-rank test. The *p* value was considered to be statistically significant at 5% level.

## Results

There were 2422 male (78.4%) and 668 female (21.6%). The median age was 58 (20–90) years. There were 370 (12.0%) well differentiated tumors, 836 (27.0%) moderately differentiated tumors and 1884 (61.0%) poorly differentiated tumors. The associations between differentiation status and clinicopathological features were summarized in Table 1. The proportion of well and moderately differentiation status were higher in patients older than 60 years old, in male patients and in patients with tumors smaller than 5 cm. It also gradually decreased along with the T stage, N stage and TNM stage. Inversely, the proportion of poorly differentiation status was higher in patients younger than 60 years old, in female patients and in patients with tumors larger than 5 cm. It also gradually increased along with T stage, N stage and TNM stage.

**Table 1:** Clinicopathological characteristics of gastric cancer patients

Characteristics	Well, *n* (%)	Moderately, *n* (%)	Poorly, *n* (%)	*P* value
Age				< 0.001
≤ 60	197(10.8)	406(22.2)	1226(67.0)	
>60	173(13.7)	430(34.1)	658(52.2)	
Gender				< 0.001
Male	308(12.7)	716(29.6)	1398(57.7)	
Female	62(9.3)	120(18.0)	486(72.7)	
Tumor location				< 0.001
Upper third	146(14.9)	351(35.7)	485(49.4)	
Middle third	47(9.3)	108(21.4)	350(69.3)	
Lower third	163(12.0)	329(24.2)	867(63.8)	
Cross or entire	14(5.7)	48(19.7)	182(74.6)	
Tumor size				< 0.001
≤ 5	332(15.3)	613(28.2)	1228(56.5)	
>5	38(4.2)	223(24.3)	656(71.5)	
Tumor depth				< 0.001
T1	193(32.9)	165(28.2)	228(38.9)	
T2	49(10.1)	153(31.5)	284(58.4)	
T3	96(8.6)	351(31.4)	670(60.0)	
T4	32(3.6)	167(18.5)	702(77.9)	
Lymph node metastasis				< 0.001
N0	274(24.2)	360(31.8)	498(44.0)	
N1	52(8.8)	200(33.8)	340(57.4)	
N2	27(5.1)	142(26.8)	361(68.1)	
N3	17(2.0)	134(16.0)	685(82.0)	
Tumor stage				< 0.001
I	225(28.9)	241(31.0)	312(40.1)	
II	93(10.1)	316(34.3)	511(55.6)	
III	52(3.7)	279(20.1)	1061(76.2)	

**Fig. 1:**
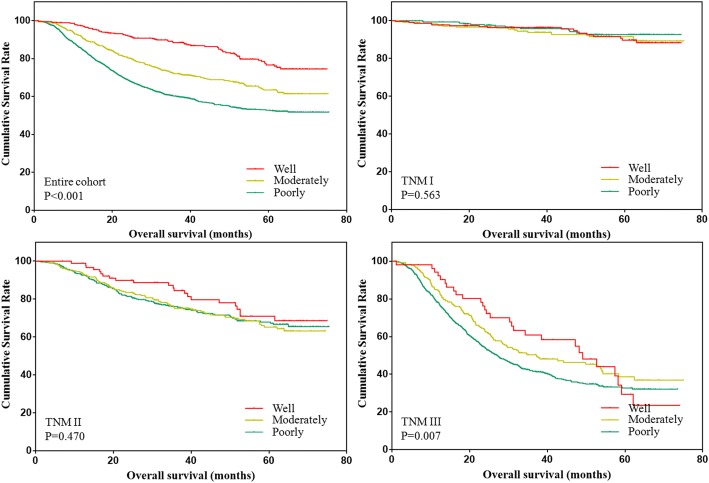
Overall survival of patients with different differentiation status

The overall survival of patients with different differentiation status were analyzed and shown in Fig. 1. The results showed that the overall survival of patients was gradually decreased along with the differentiation status. However, the difference only existed in patients with stage III tumors in the subgroup analysis.

**Fig. 2:**
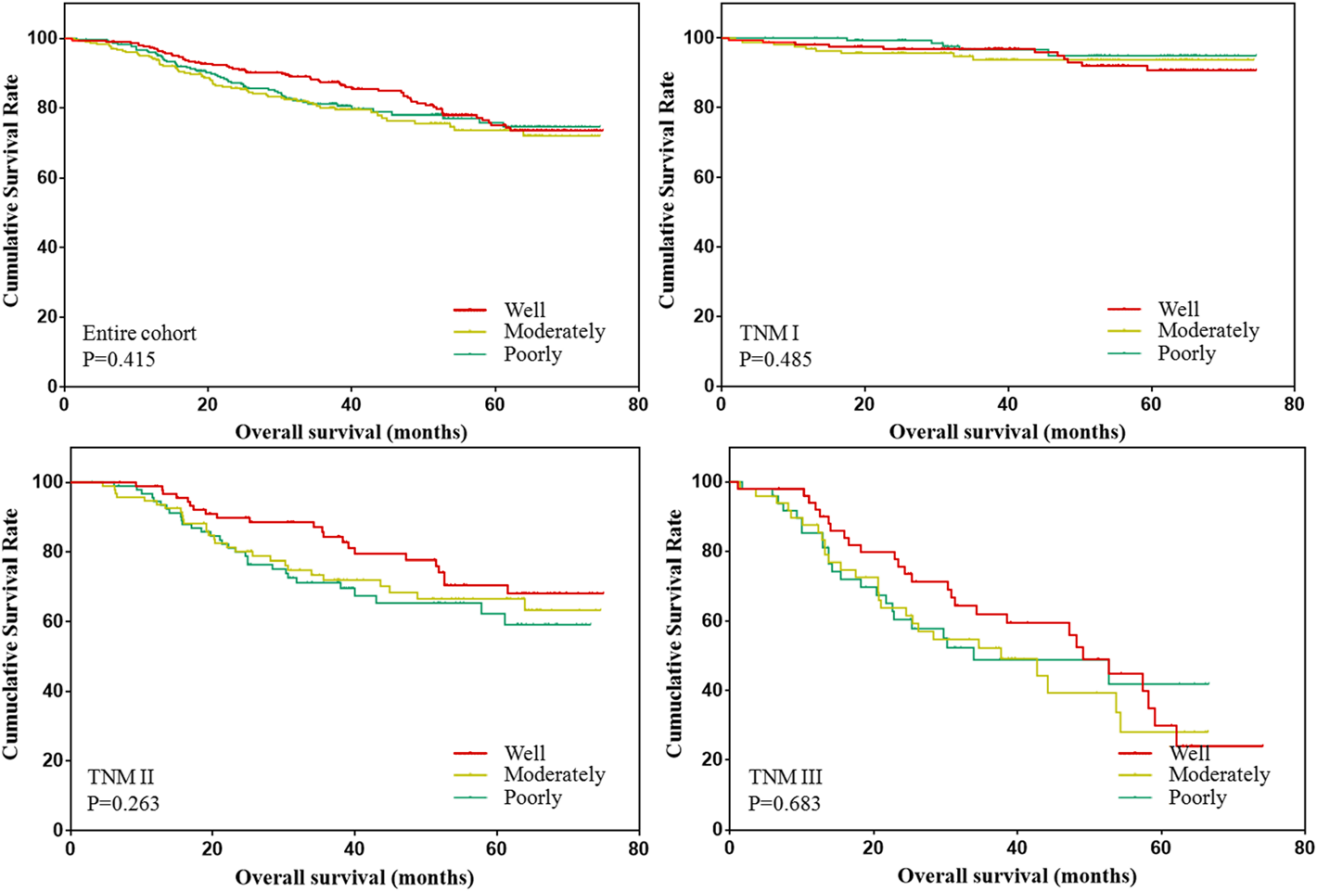
Overall survival of patients with different differentiation status after match

Due to the unbalanced baseline distribution, patients in the three groups were matched based on age, tumor size, T stage and N stage. The clinicopathological features were compared and shown in Table 2. The results showed that the distribution of age, tumor size, T stage, N stage and TNM stage were all comparable among the three groups (all *p* > 0.05). Further, the overall survival of patients was analyzed and shown in Fig. 2. The results showed that there was no significant difference among the prognosis of three groups.

**Table 2:** Clinicopathological characteristics of gastric cancer patients after match

Characteristics	Well	Moderately	Poorly	*P* value
Age				0.761
≤ 60	176	180	185	
>60	131	127	122	
Gender				0.001
Male	254	264	230	
Female	53	43	77	
Tumor location				< 0.001
Upper third	123	111	60	
Middle third	36	45	60	
Lower third	135	141	170	
Cross or entire	13	10	17	
Tumor size				0.654
≤ 5	271	270	264	
>5	36	37	43	
Tumor depth				1.000
T1	132	132	132	
T2	48	48	48	
T3	96	96	96	
T4	31	31	31	
Lymph node metastasis				1.000
N0	213	213	213	
N1	50	50	50	
N2	27	27	27	
N3	17	17	17	
Tumor stage				0.999
I	164	164	164	
II	92	94	94	
III	51	49	49

## Discussion

Up to date, the investigation of the prognostic value of differentiation status mainly focused on SRC and mucinous gastric carcinoma. Although SRC and mucinous carcinoma have long been regarded as adverse prognostic risk factors of gastric cancer, the findings of previous reports were inconsistent. This may attribute to the different sample size, race and inclusion criteria etc. in these studies. Further, in the previous reports, gastric cancer patients with SRC or mucinous differentiation status were always compared with non-SRC or non-mucinous differentiation status. The features of patients with well, moderately and poorly differentiation status have not been investigated in detail. Thus, our present study aims to elucidate the clinicopathological characteristics and prognosis of gastric cancer patients with well, moderately and poorly differentiation status. We found that differentiation status was significantly associated with age, gender, tumor location, tumor size, tumor depth, lymph node metastasis and tumor stage, but not the prognosis of gastric cancer.

In many previous reports, SRC histology appears to occur frequently in female and young patients [13, 17–19], whereas mucinous histology was not associated with gender and age of patients [10, 15, 20]. In our present study, the distribution of differentiation status was significantly associated with gender and age of patients. In detail, well and moderately differentiation status occurred more frequently in male and old patients, whereas poorly differentiation status mainly occurred in female and young patients. This was similar to the findings in patients with SRC histology in the previous reports.

Both SRC and mucinous histology were reported to be significantly associated with larger tumor size, deeper tumor invasion, more lymph node metastasis and later tumor stage in the previous reports [10, 13, 15, 20]. In our present study, we found the same tendency. Moreover, we found that the proportion of well and moderately differentiation status gradually decreased along with the T, N and TNM stage. Inversely, the proportion of poorly differentiation status gradually increased along with T, N and TNM stage. This indicated that well-moderately-poorly differentiation status transition or dedifferentiation-redifferentiation may occur in single tumor during progression of disease [21–26]. However, the potential mechanism needs further investigation.

Up to date, reports about the prognostic value of differentiation status mainly focused on SRC and mucinous carcinoma, and results were inconsistent. Piessen et al. reported that SRC histology was an independent risk factor for the poor prognosis of gastric cancer [14]. Postlewait et al. reported that SRC histology was not independently associated with reduced recurrence free survival and overall survival [13]. However, Wang et al. reported that SRC histology had a relatively favorable prognosis of early gastric cancer [12]. In addition, findings about the prognostic value of mucinous carcinoma were also controversial [10, 11, 20, 27]. The association between well, moderately and poorly differentiation status and prognosis of gastric cancer patients have never been investigated before. In our present study, differentiation status was significantly associated with the overall survival of gastric cancer patients through Kaplan-Meier analysis. However, considering the significant association between pathological features and differentiation status, patients in the three groups were matched based on age, tumor size, T stage and N stage. As a result, the overall survival was comparable between well, moderately and poorly differentiation status after match. This indicated that well, moderately and poorly differentiation status was not associated with the prognosis of gastric cancer patients.

There were several limitations in our present study. First, it was a retrospective and single center analysis with relatively limited sample size. Multi-center studies are needed to verify the findings in our present study. Second, disease free survival and disease specific survival were not analyzed. Third, only age, tumor size, T stage and N stage were matched, which may result in bias during analysis. Fourth, SRC and mucinous histology were not included in the present analysis.

## Conclusions

Well, moderately and poorly differentiation status was significantly associated with clinicopathological features of gastric cancer patients. However, it was not associated with the prognosis of gastric cancer patients.

## Abbreviations

SRC: signet ring cell
